# Vitamin D Deficiency: Monitoring and Assessment of Rehabilitation Inpatients

**DOI:** 10.1192/bjo.2025.10568

**Published:** 2025-06-20

**Authors:** Nymisha Dangeti, Joshua Wang, Sandeep Singh

**Affiliations:** Leicestershire Partnership NHS Trust, Leicester, United Kingdom

## Abstract

**Aims:** Vitamin D deficiency mainly occurs due to inadequate sun exposure and a diet insufficient in vitamin D sources. Patients undergoing rehabilitation as an inpatient have long admissions that could last years. The evidence has suggested that vitamin D deficiency is commonly observed in psychiatric inpatients and is linked to a variety of psychiatric disorders.

Aims were to evaluate the prevalence of vitamin D deficiency in a psychiatry rehabilitation unit.

To establish the treatment compliance with the current available guidance – Local Trusts and Public Health.

**Methods:** This is a re-audit of the original done in 2023 to close the loop. Data was collected for patients admitted to the psychiatric rehabilitation unit over a 3-month period in 2024. Blood tests were reviewed using the I-Lab, and treatment records were reviewed through WellSky. The data was then compared with regional standards set by the LPT (Leicestershire Partnership Trust).

**Results:** An audit conducted in 2023 involving 38 patients revealed that 5 patients (13%) had vitamin D deficiency, none of whom received replacement. Additionally, 23 patients (60.5%) out of the total 38 were administered vitamin D maintenance therapy.

A re-audit conducted in 2024, which included 35 patients, found that 3 patients (8.5%) had vitamin D deficiency, defined as a serum level below 25 nmol/L. Of those with deficiency, only 1 patient received a vitamin D replacement. Overall, 14 patients (40%) of the 35 were prescribed vitamin D supplements.

Guidelines: LPT guidance:

All mental health and learning disability inpatients to have vitamin D levels checked on admission and then annually.

Patients should be treated with standardised vitamin D replacement as needed.

All long-term (more than 3 months) inpatients should be prescribed 400 units (10 mcg) daily.

Public Health England (PHE) is advising that 10 micrograms of vitamin D are needed daily to help keep healthy bones, and muscles particularly in in autumn and winter (21 July 2016).

**Conclusion:** The earlier mentioned guidelines advise that everyone should take vitamin D supplements. However, only 40% of the patients in the re-audit were receiving the recommended supplementation.

Vitamin D deficiency is commonly observed in psychiatric patients, particularly in rehabilitation settings, due to limited sun exposure. Timely identification and treatment of vitamin D deficiency have the potential to improve patients’ mental health and prevent further deterioration of psychiatric symptoms.

